# SIRT1 haplo-insufficiency results in reduced cortical bone thickness, increased porosity and decreased estrogen receptor alpha in bone in adult 129/Sv female mice

**DOI:** 10.3389/fendo.2022.1032262

**Published:** 2022-12-07

**Authors:** Hanna Artsi, Einav Cohen-Kfir, Ron Shahar, Noga Kalish-Achrai, Natan Lishinsky, Rivka Dresner-Pollak

**Affiliations:** ^1^ Department of Endocrinology and Metabolism, Division of Medicine, Hadassah Medical Organization, Faculty of Medicine, Hebrew University of Jerusalem, Jerusalem, Israel; ^2^ Laboratory of Bone Biomechanics, Koret School of Veterinary Medicine, Faculty of Agriculture, Hebrew University of Jerusalem, Rehovot, Israel

**Keywords:** sirtuin1, estrogen receptor alpha, 129/Sv mice, microCT, femur, vertebrae

## Abstract

**Introduction:**

Sirtuin 1 (SIRT1) is a key player in aging and metabolism and regulates bone mass and architecture. Sexual dimorphism in skeletal effects of SIRT1 has been reported, with an unfavorable phenotype primarily in female mice.

**Methods:**

To investigate the mechanisms of gender differences in SIRT1 skeletal effect, we investigated femoral and vertebral cortical and cancellous bone in global Sirt1 haplo-insufficient 129/Sv mice aged 2,7,12 months lacking *Sirt1* exons 5,6,7 (*Sirt1^+/Δ^*) and their wild type (WT) counterparts.

**Results:**

In females, femoral bone mineral content, peak cortical thickness, and trabecular bone volume (BV/TV%), number and thickness were significantly lower in *Sirt1^+/Δ^* compared to WT mice. Increased femoral cortical porosity was observed in 7-month-old *Sirt1^+/Δ^* compared to WT female mice, accompanied by reduced biomechanical strength. No difference in vertebral indices was detected between *Sirt1^+/Δ^* and WT female mice. SIRT1 decreased with aging in WT female mice and was lower in vertebrae and femur in 18- and 30- versus 3-month-old 129/Sv and C57BL/6J female mice, respectively. Decreased bone estrogen receptor alpha (ERα) was observed in *Sirt1^+/Δ^* compared to WT female mice and was significantly higher in *Sirt1* over-expressing C3HT101/2 murine mesenchymal stem cells. In males no difference in femoral indices was detected in *Sirt1^+/Δ^* versus WT mice, however vertebral BV/TV%, trabecular number and thickness were higher in *Sirt1^+/Δ^* vs. WT mice. No difference in androgen receptor (AR) was detected in bone in *Sirt1^+/Δ^* vs. WT male mice. Bone SIRT1 was significantly lower in male compared to female WT mice, suggesting that SIRT1 maybe more significant in female than male skeleton.

**Discussion:**

These findings demonstrate that 50% reduction in SIRT1 is sufficient to induce the hallmarks of skeletal aging namely, decreased cortical thickness and increased porosity in female mice, highlighting the role of SIRT1 as a regulator of cortical bone quantity and quality. The effects of SIRT1 in cortical bone are likely mediated in part by its regulation of ERα. The age-associated decline in bone SIRT1 positions SIRT1 as a potential therapeutic target to ameliorate age-related cortical bone deterioration in females. The crosstalk between ERα, AR and SIRT1 in the bone microenvironment remains to be further investigated.

## Introduction

Sirtuin 1 (SIRT1), a NAD^+^-dependent deacetylase, plays a key role in aging and metabolism (1–3). We and others have previously shown that SIRT1 regulates bone mass and micro-architecture *via* its direct effects on various cell types in the bone microenvironment. Using mouse models of global *Sirt1* overexpression as well as global and cell specific *Sirt1* deletion in bone marrow mesenchymal stem cells, pre-osteoblasts, osteoblasts, and osteoclasts it has been shown that SIRT1 stimulates bone formation and inhibits bone resorption and marrow adipogenesis (4–8). Key factors in bone homeostasis such as sclerostin, a canonical WNT pathway inhibitor, RUNX2, FOXOs, PPARγ, NFĸB, PGC1α, and β-catenin were identified as SIRT1 targets (9–11).

Sexual dimorphism in SIRT1 effect in bone has been previously reported by us and others with a compromised phenotype observed in *Sirt1* deficient female but not male mice (4, 6). To understand the underlying mechanisms of gender differences in SIRT1 skeletal effect, we investigated bone microarchitecture and biomechanical strength in 129/Sv *Sirt1^+/Δ^* female and male mice and their WT littermates. We discovered that *Sirt1* haplo-insufficiency differentially affects cortical and trabecular bone accrual in female and male mice, while SIRT1 directly upregulates estrogen receptor type alpha (ERα) in bone. Adult *Sirt1^+/Δ^* female mice displayed reduced femoral cortical thickness and increased porosity, the hallmarks of skeletal aging, at an early age of age 7 month, accompanied by biomechanical deterioration. A higher bone SIRT1 level was found in female compared to male WT mice with a dramatic decline with aging in two different mouse strains, suggesting that SIRT1-based therapeutics maybe beneficial for age-associated cortical bone deterioration in females.

## Methods

### Animal experimentation

Adult inbred female and male S*irt1* haplo-insufficient mice (*Sirt1^+/Δ^*) and their wild type (WT) littermates of 129/Sv background (12) were a generous gift of Prof. Frederick W. Alt of Harvard University and were previously studied by us (4, 8). Two mouse models of *Sirt1* deficient 129/Sv mice have been generated: *Sirt1^Δex4^* lacking the 4^th^ exon that encodes for the conserved SIRT1 catalytic domain, and *Sirt1^Δneo^* lacking exons 5, 6 and 7 resulting in no SIRT1 protein generation. General *Sirt1* ablation in both mouse models resulted in a high degree of post-natal lethality with less than 5% of mice surviving to adulthood. Knock-out mice are small in body size and exhibit significant developmental defects of the retina and the heart (12).

Mice were housed under specific pathogen free (SPF) conditions with free access to water and chow #2018 (Teklad Diets, Madison WI) containing 18.6% protein, 1% calcium and 2 IU/g vitamin D_3_ and water. Three- and 30-month-old C57BL/6J female mice were obtained from the National Institute of Aging (NIA) through scientific collaboration with Prof. Raul Mostoslavsky of Harvard University. Upon sacrifice by CO_2_ inhalation blood was collected *via* cardiac puncture, immediately separated and frozen in -80°C until used. Femurs and L4 were removed, cleaned of adherent tissue, kept in 10% formalin for 48 hours and then in 70% EtOH at 4°C for micro-computed tomography (μCT) imaging. For biomechanical testing femurs were immediately wrapped in saline-soaked gauze and kept in -20°C until analyzed. Vertebrae, femurs, and tibiae were collected for protein and RNA extraction and stored at -80°C until analyzed. Bone marrow from femurs and tibiae was flushed and lysed as described below. All experiments were performed with the approval of the Animal Study Committee of the Hebrew University-Hadassah Medical School (MD-12-13154-3). All studies were conducted in accordance with ARRIVE guidelines.

### µCT analyses

L4 and femurs of female and male *Sirt1^+/Δ^* and WT mice aged 2, 3, 7 and 12 months were examined *ex-vivo* by μCT (Desktop µCT 42; Scanco, Switzerland). Each group of *Sirt1^+/Δ^* and WT mice was studied separately. The scanner was operated at X-ray tube potential 70 kVp and X-ray intensity 114 µA, with an integration time of 200 ms and isotropic resolution of 10 µm. Femoral length was automatically measured in μCT images. Trabecular bone was analyzed in L4, and in the secondary spongiosa in the distal femoral metaphysis. Cortical bone was analyzed in the femoral mid-shaft. Cortical porosity was assessed as the percentage of void area out of cortical bone 1 mm distal to the midshaft. The chosen area was contoured, and threshold set to 280 units (588 mg HA/ccm) for the midshaft evaluation 210 units (390 mg HA/ccm) for the trabecular structure. Evaluation was performed by running the built-in “eval_midshaft” script with uct_evaluation_v6 software version (Scanco).

### Biomechanical testing

Femora were subjected to the three-point bending test. Force–displacement data was generated as previously described (13). Monotonic loading was performed at a constant rate of 250 μm/min. Force to fracture (breaking force), and maximal force (ultimate force) were obtained from the load-displacement curves. Ultimate stress *σ*_*u*_ as calculated based on the µCT and force-displacement data using the formula: $\sigma_{u}=\frac{F_{u}Lc}{4I}$ , F_u,_ ultimate force, L, the distance between the support points (5 mm) c, half-width of mid-shaft in the load direction derived from the µCT measurements, I, cross sectional moment of inertia (CSMI) (14).

### Experiments in the mesenchymal stem-cell line C3H10T1/2

*Sirt1* over-expression in the murine mesenchymal embryonic fibroblast stem cell line C3H10T1/2 (ATCC CCL-226) was previously modified by us through retroviral infection with pBABE-*Sirt1* (4). *Sirt1* over-expressing and control C3H10T1/2 cells were plated in growing medium (GM; D-MEM/10% fetal calf serum/2 mM L-Glutamine/100 Units/ml penicillin/100 mg/ml streptomycin sulfate/0.25 mg/ml amphotericin B) and were maintained for 4 days for immunoprecipitation and 14 days for studying protein and mRNA expression.

### Protein analysis

Protein from whole vertebrae and femora was extracted by crushing the bones in liquid nitrogen followed by lysis in RIPA buffer (50mM Tris pH7.5/150 mM NaCl/0.1% SDS/0.5% sodium deoxycholate/1% Triton X 100) and additional crushing by Polytron (Kinematica). For C3HT101/2 cells protein was extracted in Laemmli buffer (2% SDS/10% glycerol/5% 2-mercaptoethanol/0.01% bromphenol blue/60 mM Tris HCl). Nuclear extracts of bone marrow cells flushed from tibiae and femora were obtained by using the nuclear Extraction Kit # 10009277 (Cayman Chemicals). Antibodies for immunoblotting: SIRT1 (Millipore, 07-131), αTubulin (AbCam, ab106375), HSP90 (Heat Shock Protein 90) (BD Transduction laboratories), ERα (Estrogen receptor alpha) (AbCam, ab2746), Histone 3 (AbCam, ab1791), Acetylated-Lysine (Cell Signaling, C-9441L), Androgen receptor (Millipore, MM06680), GAPDH (Glyceraldehyde-3-phosphate dehydrogenase) (AbCam, ab8245). Quantification for Western blot images was performed by using a digital camera, BIO-RAD CHEMIDOC using the software IMAGELAB and IMAGEJ.

### Immunoprecipitation

*Sirt1* over-expressing and control C3HT101/2 cells were lysed in lysis buffer (50mM TRIS-HCl, pH 7.4/1% NP-40, 0.25% Sodium deoxycholate/150 mM NaCl, 1mM EDTA/1mM PMSF supplemented with protease inhibitor and phosphatase inhibitor). Marrow from tibiae and femora was flushed with the same buffer, then cleared by centrifugation at 12,000 RPM for 15 min at 4°C. Immunoprecipitation was carried out by preclearing 1 μg of protein in 300 μl lysis buffer with 30 μl protein A beads (Millipore) for 1 h and incubating the lysates with 4 μl of the SIRT1 antibody (Millipore, 07-131), rotating over night at 4°C. For precipitation, 30 μl protein A agarose beads were added followed by incubation at 4°C for 3 h. Immunoprecipitates were washed extensively and eluted twice with x2 Laemmli buffer. Proteins were separated by SDS-PAGE and transferred to PVDF membranes (Millipore).

### Gene expression analysis

Whole vertebrae and C3H10T1/2 cells were homogenized in TRIzol (Invitrogen, Carlsbad CA). Total RNA was extracted and converted to cDNA using the qScript kit (Quanta BioSciences, Inc. Gaithersburg, MD, USA). Gene expression analysis was performed using SYBR Green-based real-time-PCR (Kapa Syber, Kapa Biosystems (Pty) Ltd, Cape Town, South Africa). Relative gene expression was determined by the comparative cycle threshold (CT) method with *βActin, GAPDH* and *Polr2A* as controls. For each sample, the mean CT for each gene (run in triplicate) was normalized to the geometric mean of the mean CT of the 3 reference genes using the formula: 2^-(gene of interest CT-reference CT)^. The resulting ΔCT for each gene was used to calculate relative gene expression changes between samples.

### Statistical analysis

Statistical analysis was performed with GraphPad Prism 9.3.1 statistical software (GraphPad Software, Inc., La Jolla, CA, USA). All data were tested for normality using the Shapiro-Wilk test. Normally distributed data sets were analyzed with parametric tests, whereas data sets that did not pass the Shapiro-Wilk test (*P*<0.05) were analyzed with nonparametric tests. For normally distributed data, unpaired 2-tailed Student’s *t* test was used to compare means of two groups. µCT metrics were compared in 2, 3, 7 and 12-month-old female and male mice and their age-matched WT counterparts using the unpaired 2-tailed Student’s *t* test. Data are presented as Mean ± SEM. Differences of *P*< 0.05 were considered significant.

## Results

### Decreased femoral peak cortical thickness and bone volume fraction in Sirt1^+/Δ^ female but not male mice

Body weight was mildly reduced in *Sirt1^+/Δ^* compared to WT female mice at age 2 months but similar in both genotypes beyond that age (**Supplementary Figure S1A**). In males, no difference in body weight could be detected between genotypes (**Supplementary Figure S1B**). Femoral length was lower in *Sirt1^+/Δ^* compared to WT female mice at age 2 months but similar in both genotypes at age 7 months (**Supplementary Figure S2A**). Of note, we have previously reported similar serum IGF-1 level in this model of *Sirt1^+/Δ^* and WT female mice (4). In males, no difference in femoral length could be detected between genotypes (**Supplementary Figure S2B**).

**Figure 1:**
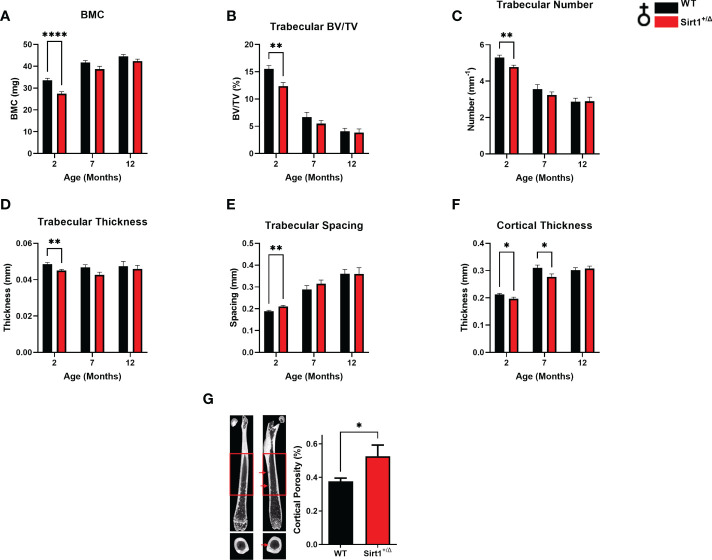
*Sirt1* haploinsufficiency leads to reduced peak cortical thickness, decreased trabecular bone volume fraction and increased cortical porosity in the femur in *129/Sv* female mice. µCT analysis of femoral mid-shaft and distal metaphysis in 2,7,12-month-old *Sirt1^+/Δ^* and WT female mice. **(A)** Bone mineral content (BMC) **(B)** Bone volume fraction (BV/TV%) **(C)** Trabecular number **(D)** Trabecular thickness **(E)** Trabecular spacing **(F)** Cortical thickness **(G)** Cortical porosity percent in 7-month-old *Sirt1^+/Δ^* and WT female mice (n = 14-15 mice/group); representative image (left) and quantification (right); scale bar 1 mm. Results are Mean ± SEM analyzed by unpaired Student’s *t* test. *P < 0.05, **P < 0.01, ****P < 0.0001 versus WT female mice. ((n = 7-11 mice/group). BV, bone volume; TV, total volume.

**Figure 2:**
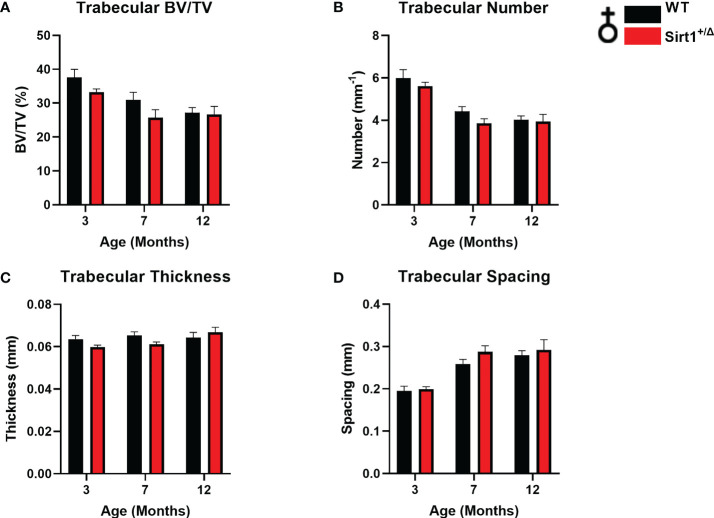
The effects of *Sirt1* haploinsufficiency on vertebral L4 trabecular bone indices in female mice. µCT analysis of L4 trabecular bone in 3, 7,12-month-old *Sirt1^+/Δ^* and WT female mice. **(A)** Bone volume fraction (BV/TV%) **(B)** Trabecular number **(C)** Trabecular thickness **(D)** Trabecular spacing. Results are Mean ± SEM analyzed by unpaired Student’s *t* test (n = 7-11 mice/group).

Femoral bone mineral content (BMC) was significantly lower in *Sirt1^+/Δ^* compared to WT female mice at age 2 months (**Figure 1A**). Femoral trabecular peak bone mass expressed as bone volume fraction (BV/TV%) was attained at age 2 month in both genotypes and was significantly lower in *Sirt1^+/Δ^* compared to WT female mice (**Figure 1B**). Consistently, reduced trabecular number and thickness accompanied by increased trabecular spacing was observed in 2-month-old *Sirt1^+/Δ^* compared to WT female mice (**Figures 1C–E**). Cortical thickness peaked at age 7 month in both *Sirt1^+/Δ^* and WT female mice and was significantly lower in *Sirt1^+/Δ^* compared to WT mice at both age 2 and 7 months (**Figure 1F**). Importantly, a 50% increase in cortical porosity percent was found in femoral midshaft in *Sirt1^+/Δ^* compared to WT female mice at age 7 months (**Figure 1G**). Peak vertebral (L4) BV/TV% was attained at age 3 months in female mice in both genotypes (**Figure 2A**) and was not significantly different in *Sirt1^+/Δ^* versus WT female mice, nor were other indices of vertebral trabecular bone (**Figures 2B–D**).

### Increased vertebral trabecular bone volume fraction and number in Sirt1^+/Δ^ male but not female mice

**Figure 3:**
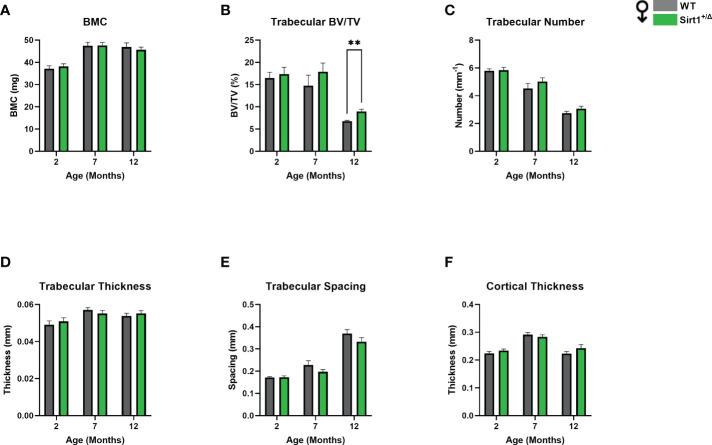
The effects of *Sirt1* haploinsufficiency on femoral cortical and trabecular bone parameters in *Sirt1^+/Δ^* and WT male mice. µCT analysis of femoral mid-shaft and distal metaphysis in 2, 7,12-month-old *Sirt1^+/Δ^* and WT male mice. **(A)**. Bone mineral content (BMC) **(B)**. Bone volume fraction (BV/TV%) **(C)**. Trabecular number **(D)**. Trabecular thickness **(E)**. Trabecular spacing **(F)**. Cortical thickness in mid shaft. Results are Mean ± SEM analyzed by unpaired Student’s *t* test; **P < 0.01 versus WT male mice. (n = 7-11 mice/group).

**Figure 4:**
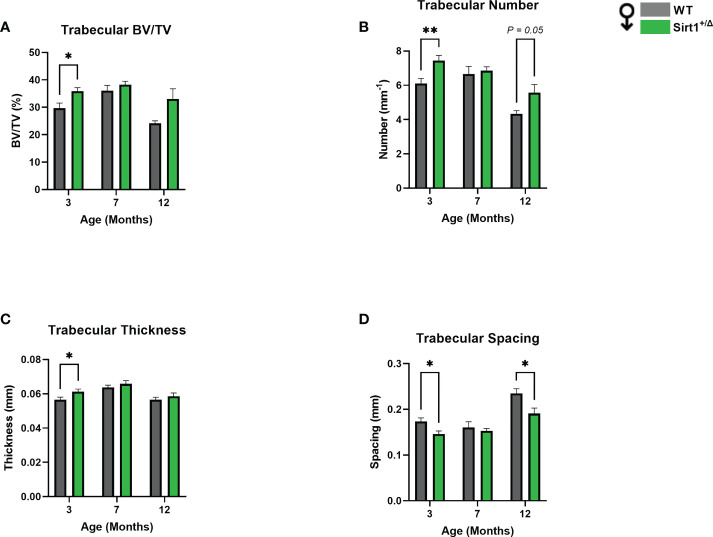
*Sirt1* haploinsufficiency leads to increased vertebral L4 trabecular bone indices in *Sirt1^+/Δ^* male mice. µCT analysis of L4 trabecular bone: **(A)** Bone volume fraction (BV/TV%) **(B)** Trabecular number **(C)** Trabecular thickness **(D)** Trabecular spacing. Results are Mean ± SEM analyzed by unpaired Student’s *t* test; *P < 0.05, **P < 0.01 versus WT male mice (n = 7-11 mice/group).

A different phenotype was observed in male mice. Like in females, cortical thickness peaked at age 7 months in both genotypes (**Figure 3F**), however no difference in BMC or cortical thickness was detected between WT and *Sirt1^+/Δ^* male mice (**Figures 3A, F**). Femoral BV/TV% was higher in *Sirt1^+/Δ^* versus WT male mice at age 12 months (**Figure 3B**). Strikingly, vertebral L4 BV/TV%, trabecular number and thickness were significantly higher in 3-and 12-month-old *Sirt1^+/Δ^* compared to WT male mice (**Figures 4A–C**).Consistently, trabecular spacing was lower in *Sirt1^+/Δ^* compared to WT male mice (**Figure 4D**). Taken together, these results show a gender dimorphic effect of *Sirt1* haplo-insufficiency on femoral cortical and cancellous and vertebral cancellous bone with lower femoral indices in *Sirt1^+/Δ^* versus WT female mice, and higher vertebral indices in *Sirt^1+/Δ^* versus WT male mice.

### Decreased femoral biomechanical strength in Sirt1^+/Δ^ female but not male mice

**Figure 5:**
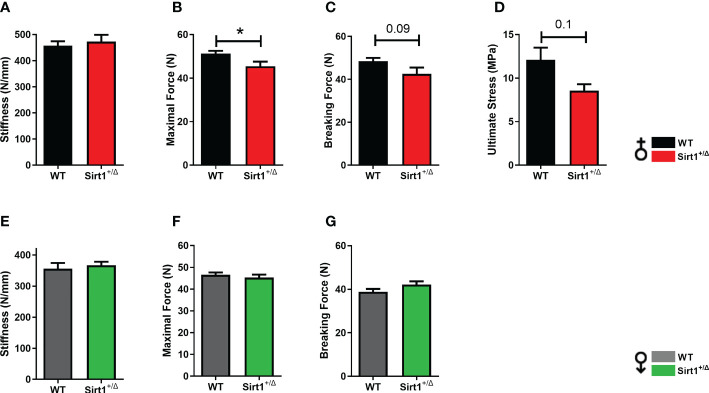
*Sirt1* haploinsufficiency leads to reduced femoral biomechanical properties in female but not male *Sirt1^+/Δ^* mice. Biomechanical properties determined by three-point bending in 7-month-old female **(A–D)** and male **(E–G)** *Sirt1^+/Δ^* and WT mice. **(A, E)**. Stiffness (N/mm). **(B, F).** Maximal (Ultimate) force (N). **(C, G)**. Breaking force (N). **(D)** Ultimate Stress (MPa). Results are mean ± SEM. Analyzed by unpaired Student’s *t*-test. *P < 0.05 versus WT mice. (n = 8 mice/group).

To understand if reduced femoral cortical thickness and increased porosity in *Sirt1^+/Δ^* female mice result in biomechanical alterations, the three-point bending test was performed in *Sirt1^+/Δ^* female and male mice and their WT counterparts. (**Figures 5A–G**). While stiffness was not affected (**Figure 5A**), maximal force was significantly reduced in *Sirt1^+/Δ^* compared to WT female mice (**Figure 5B**). There was also a trend for lower breaking force (**Figure 5C**) *(P=0.09)* and ultimate stress (**Figure 5D**) (*P=0.1*). No difference in any of these parameters could be detected in *Sirt1^+/Δ^* compared to WT male mice (**Figures 5E–G**), suggesting that femoral mechanical strength is reduced in female but not male *Sirt1^+/Δ^* mice.

### SIRT1 upregulates ERα in bone

**Figure 6:**
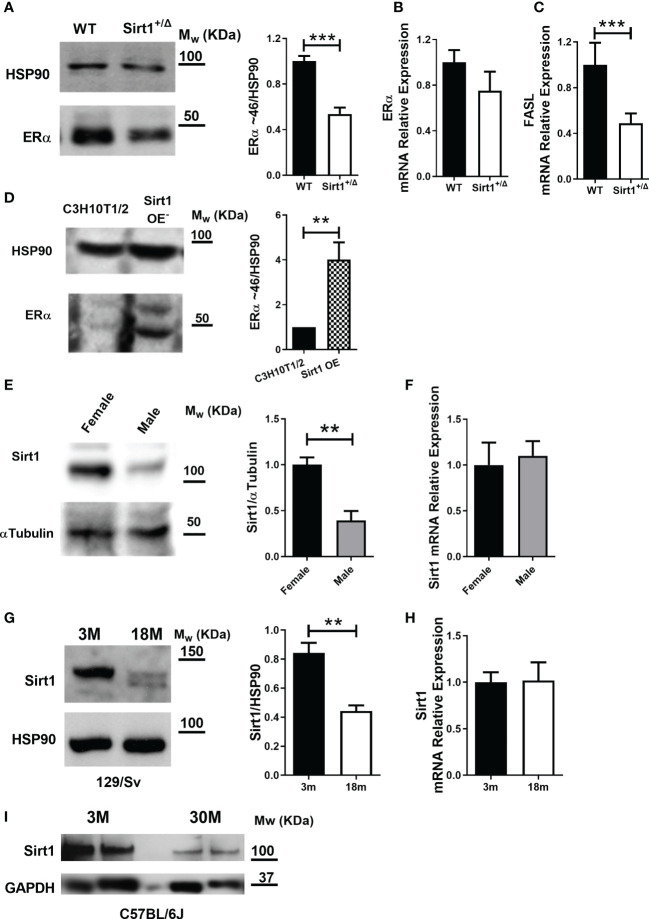
SIRT1 upregulates ERα in bone, is lower in males and declines with aging in female mice. **(A)** Immunoblot of ERα in whole vertebrae in 5-month-old *Sirt1^+/Δ^* and WT female mice. A representative image (left) and densitometry (right) with HSP90 as control. **(B)** *ERα* mRNA and **(C)** *FASL* mRNA relative expression in whole vertebrae in 5-month-old *Sirt1^+/Δ^* and WT female mice with *βActin* and *GAPDH* as controls. **(D)** Immunoblot of ERα in *Sirt1* over-expressing and control C3HT101/2 cells. A representative image (left) and densitometry (right) with HSP90 as control (n = 3 independent experiments). **(E)** SIRT1 protein and **(F)** *Sirt1* mRNA in whole vertebrae in 5-month-old WT female and male mice. A representative image (left) and densitometry (right) with α-tubulin and *GAPDH* as controls, respectively. **(G)** SIRT1 protein level in whole vertebrae in 3- and 18-month-old WT 129/Sv female mice (n = 3 mice/group). A representative image (left) and densitometry (right) with HSP90 as control. **(H)** *Sirt1* mRNA relative expression in whole vertebrae in 3- and 18-month-old WT 129/Sv female mice (n = 3 mice/group) with *βActin*, *Polr2A* and *GAPDH* as controls. **(I)** SIRT1 protein level in whole femur in 3- and 30-month-old C57BL/6J WT female mice (n = 3 mice/group) with GAPDH as control. Results are mean ± SEM analyzed by unpaired Student’s *t*-test. **P < 0.01 versus C3H10T1/2 cells **(D)**; **P < 0.01 versus WT female mice **(E)**; **P < 0.01 versus 3-months-old WT 129/Sv female mice; **(G)** ***P < 0.001 versus WT female mice **(A, C)** (n = 6-9 mice/group, unless otherwise specified).

To gain insight into possible underlying mechanisms of gender differences in the effects of *Sirt1* haplo-insufficiency on cortical and cancellous bone, we next sought to explore sex hormone receptors in bone in *Sirt1^+/Δ^* and WT female and male mice. We have previously reported that serum E_2_ levels were not different in *Sirt1^+/Δ^* and WT female mice (4). Using the ALPCO Mouse/Rat Testosterone ELISA assay to determine serum testosterone, levels were under the detection level of the kit in *Sirt1^+/Δ^* and WT male mice (data not shown). ERα plays a key role in bone in female and male mice as well as humans (15). ERα deletion from early osteoblast progenitors (16) or mature osteoblasts (17–20) was previously shown to result in decreased cortical bone mass in female but not male mice. We therefore evaluated ERα in *Sirt1^+/Δ^* and WT female mice. Strikingly, ERα protein level was dramatically reduced by approximately two-fold in whole vertebrae extracts derived from *Sirt1^+/Δ^* compared to WT female mice (**Figure 6A**). mRNA expression was only mildly reduced (**Figure 6B**). Consistently, mRNA expression of Fas ligand (*FASL)*, an ERα target gene in osteoblasts (21), was decreased in vertebrae obtained from *Sirt1^+/Δ^* compared to WT female mice (**Figure 6C**). To further investigate if the effects of Sirt1 on ERα are direct and cell autonomous, we compared ERα expression in *Sirt1*-overexpressing CH310T1/2 mesenchymal stem cells and control cells. Consistent with the *in vivo* findings, ERα protein and mRNA levels were markedly increased in *Sirt1* over-expressing C3H10T1/2 (**Figure 6D**), indicating upregulation of ERα induced by SIRT1. This data suggest that SIRT1 upregulates ERα in bone and its reduction in *Sirt1^+/Δ^* female mice likely contributes to reduced femoral bone indices in female mice.

### Bone SIRT1 is lower in male compared to female WT 129/Sv mice

Others and we have previously shown that estrogen deficiency induced by ovariectomy results in reduced SIRT1 in bone (5, 22), suggesting that SIRT1 is regulated by estrogens. We therefore compared SIRT1 protein level in vertebral extracts obtained from male and female WT mice. Strikingly, a marked reduction in SIRT1 protein was observed in male compared to female WT mice (**Figure 6E**). No difference was observed in *Sirt1* mRNA expression (**Figure 6F**). It is therefore plausible that no significant SIRT1-dependent deterioration in cortical bone phenotype was observed in male mice due to significantly lower SIRT1 level in male bone.

### SIRT1 decreases with aging in bone in 129/Sv and C57BL/6J WT female mice

To understand the relevance of these findings to skeletal aging in female mice, we compared SIRT1 protein level in spine and femur in young versus aged WT female mice in two mouse strains. Vertebral SIRT1 level was markedly reduced in 18-month- compared to 3-month-old 129/Sv WT female mice (**Figure 6G**). Consistently, SIRT1 was lower in femora in 30-compared to 3-month-old C57BL/6J female mice (**Figure 6I**), confirming that the effect of aging on bone SIRT1 is not strain-dependent.

### SIRT1 and androgen receptor

**Figure 7:**
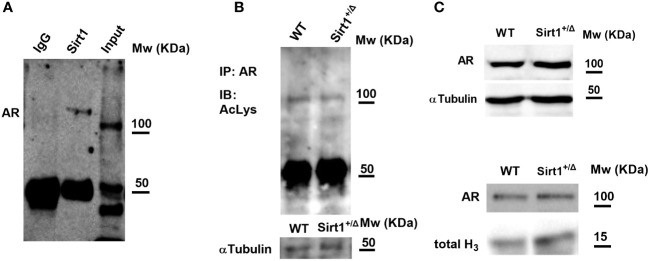
SIRT1 and the androgen receptor (AR). **(A)** SIRT1 associates with AR in *Sirt1*-over expressing C3HT101/2 cells; immunoprecipitation (IP) with anti SIRT1 and IgG (negative control) antibodies. **(B)** AR acetylation in bone marrow flush in 9-week-old *Sirt1^+/Δ^* and WT male mice. IP with anti SIRT1 antibody, immunoblot with anti-acetylated lysine antibody. **(C, D)**. AR in bone marrow flush in 9-week-old *Sirt1^+/Δ^* and WT male mice; C-Cytosolic fraction with α-tubulin as control. D-Nuclear fraction with Histone 3 (H3) as control (a pool of 3 mice/group).

To explore the underlying mechanisms of increased vertebral cancellous bone indices in *Sirt1^+/Δ^* vs. WT male mice, we sought to investigate the AR, as SIRT1 was previously reported to deacetylase and inhibit AR function in prostate cells in the context of prostate cancer (23). Thus, *Sirt1* haplo-insufficiency could result in restraining the inhibitory effects of SIRT1 on AR. Furthermore, targeted deletion of exon 3 of the AR in mature osteoblasts resulted in reduced vertebral cancellous bone volume fraction and trabecular number (24). We, therefore, first examined if AR and SIRT1 physically interact with each other in *Sirt1* overexpressing C3H10T1/2 cells. A physical interaction between SIRT1 and AR was observed (**Figure 7A**). Next, we determined lysine acetylation, a marker of SIRT1 activity, in bone marrow flush obtained from *Sirt1^+/Δ^* and WT male mice. No difference in AR lysine acetylation could be detected in WT versus *Sirt1^+/Δ^* male mice (**Figure 7B**), nor was there a difference in AR protein level in the cytosolic or the nuclear fractions obtained from long bones marrow flush in *Sirt1^+/Δ^* vs. WT male mice (**Figures 7C, D**).

## Discussion

This study demonstrates that global *Sirt1* haplo-insufficiency attenuates femoral cortical and trabecular bone mass accrual in female but not male 129/Sv mice, resulting in lower femoral bone mineral content, a corelate of human bone mineral density, decreased peak femoral cortical bone thickness and metaphyseal cancellous bone volume fraction. Moreover, increased cortical porosity was observed in femoral cortical bone in *Sirt1* haplo-insufficient female mice at a relatively young age of 7 months. Consistent with the notion that cortical porosity is a major determinant of bone strength (25, 26) these structural alternations in adult *Sirt1^+/Δ^* female mice resulted in deterioration in femoral biomechanical strength, as indicated by lower maximal and breaking forces. Thus, adult *Sirt1^+/Δ^* female mice prematurely display the hallmarks of skeletal aging namely reduced cortical thickness and increased cortical porosity. As peak bone mass is a predictor of bone quality at old age (27), and cortical porosity is a structural deterioration associated with increased fracture risk in mice and humans, our findings highlight the importance of SIRT1 in skeletal aging in female mice. Importantly, we discovered that SIRT1 expression in vertebrae and femur significantly declines with age in female mice in two different mouse strains, supporting the notion that the physiologic decline in SIRT1 is a significant contributor to age-associated cortical bone deterioration in females. Of note, in humans, porosity occurs in both genders but is much higher in women compared to men (28, 29).

To investigate the underlying mechanisms of sexual dimorphism in the skeletal effects of *Sirt1* haplo-insufficiency, we studied sex hormone receptors, as a cross talk between SIRT1 and ERα and AR has been previously reported in the context of breast and prostate cancer, respectively (23, 30). Indeed, reduced vertebral ERα was found in *Sirt1^+/Δ^* female mice, while increased ERα was observed in Sirt1 over-expressing C3H10T1/2 cells indicating direct upregulation of ERα. This data is consistent with the results of Yao et al. (31) that have shown decreased ERα protein level in mouse embryonic fibroblast (MEF) cells derived from *Sirt1^-/-^* mice.

The interaction between sex hormone receptors and bone is complex, and sex hormone receptors were previously shown to regulate bone mass and microstructure in a gender specific manner (32). ERα plays a key role in bone in both females and males in mice and humans. Studies investigating the effects of ERα actions on bone have used mouse models with global as well as cell specific ERα deletion in different bone cells in bone including osteoprogenitors, osteoblasts, osteocytes, osteoclasts, and immune cells. The findings in global ERα deletion mouse models were confounded by the systemic increase in circulating sex hormones, and models of targeted ERα deletions from birth onwards could affect growth and development. With these limitations, in most studies a gender specific skeletal phenotype was observed. Targeted deletion of ERα in myeloid progenitors resulted in cancellous bone loss in female but not male mice (33). Using the Prx-1-Cre mouse model to delete ERα in mesenchymal progenitors led to reduced cortical bone mass in female but not male mice (16). Similarly, ERα deletion in osteoblasts progenitors using the Osx1-Cre model (16) and in osteoblasts using the OCN-Cre model also resulted in reduced cortical bone mass in female but not male mice (34). Importantly, in a recently published study in which ERα deletion in osteocytes was induced in adult mice using the tamoxifen inducible CreERT2 with the 8kb Dmp1 promoter, it was shown that ERα is critical for estrogen action in adult bone in female but not male (15). Taken together, we speculate that reduced SIRT1 resulted in lower ERα in bone and contributed to impaired cortical bone phenotype in *Sirt1^+/Δ^* female mice. As SIRT1 level was found in this study to be significantly lower in males, the change in ERα in males was probably marginal. Interestingly, the orphan receptor estrogen related receptor alpha (ERRα) was also reported to influence cortical bone (35) and be regulated by SIRT1 (36).

On the other hand, AR was previously shown to regulate primarily cancellous bone in male mice. Targeted deletion of AR in the mesenchymal lineage and in osteoblasts using the 2.3kb Col1A1 mouse model resulted in low cancellous bone but not cortical bone mass in male but not female mice (24). Of note, transgenic mice overexpressing AR in osteoblasts under the control of the 2.3-kb α1 (I)-collagen promoter fragment exhibited increased trabecular bone volume and trabecular number (37). As *Sirt1^+/Δ^* male mice exhibited higher vertebral BV/TV% and previous data has shown inhibition of AR by SIRT1, we anticipated a decrease in AR level or function. However, we could not detect differences in AR expression in whole vertebrae in *Sirt1^+/Δ^* compared to WT male mice. Additional studies in different cell types and bone compartments are needed to fully understand the interaction between AR and SIRT1 in bone.

Lower SIRT1 level was observed in male compared to female mice. These findings are consistent with Elangovan et al. (30) that examined *Sirt1* mRNA expression in kidney, lung, liver, heart, and colon in C57BL/6J female and male mice and found lower *Sirt1* expression in males compared to females aged 3-6 months. Bone tissue was not included in that study.

This study is not without limitations. We used a mouse model with global *Sirt1* haplo-insufficiency. Thus, the skeletal phenotype observed in this study could result from direct actions of SIRT1 in bone cells as well as indirect effects in other tissues and *via* intermediate mediators. SIRT1 plays a role in skeletal muscle physiology and reduction in muscle mass and function can contribute to bone loss. SIRT1 positively regulates peroxisome proliferator-activated receptor γ coactivator 1α (PGC-1α), a major inducer of mitochondrial biogenesis and the expression of antioxidative enzymes, that can inhibit the generation of harmful mitochondrial reactive oxygen species (ROS) (38). In addition, SIRT1 influences the activity of the transcription factors forkhead box, class O (FoxO), FoxO1 and FoxO3 in muscle, thereby inhibiting muscle atrophy and promoting muscle growth (39). Investigating skeletal phenotypes in mouse models of targeted *Sirt1* deletion in muscle cells can shed light on additional pathways by which SIRT1 exerts its effects in bone. Gender differences in bone SIRT1 level were evaluated in 129/Sv mice and not in additional mouse strains, although we demonstrated age-associated reduction in bone SIRT1 in two different mouse strains. The effects of Sirt1 on ERα and AR were studied in whole vertebrae at one time point and not in specific bone cells in the different bone compartments. It is possible that we could not detect differences in AR in *Sirt1^+/Δ^* compared to WT male mice due to the confounding effects of a mixed cell population. ERβ was not studied as its skeletal effects were shown to play a less significant role in bone homeostasis as the skeletal phenotype of mice with global deletion of ERβ is minimal (40). Additional studies investigating the crosstalk between sex hormones, their receptors and SIRT1 are needed to increase our understanding of SIRT1-related gender dimorphic effects in bone.

In conclusion, our results indicate that *Sirt1* haplo-insufficiency in adult female 129/Sv mice leads to reduced peak femoral cortical thickness and trabecular bone volume fraction, as well as increased cortical porosity, accompanied by unfavorable biomechanical properties and decreased bone ERα expression. The notion that reduction of SIRT1 level by half is sufficient to induce cortical porosity in females at adulthood identifies SIRT1 as a regulator of cortical bone quantity and quality, and positions SIRT1 as a potential target to improve cortical bone mass and strength. Whether a feedback mechanism exists between sex hormones, ERα, AR and SIRT1 that contributes to the sexual dimorphism detected in SIRT1 skeletal effects remains to be further investigated.

## Data availability statement

The raw data supporting the conclusions of this article will be made available by the authors, without undue reservation.

## Ethics statement

The animal study was reviewed and approved by Animal Study Committee of the Hebrew University-Hadassah Medical School (MD-12-13154-3).

## Author contributions

HA designed the experiments and performed the μCT analyses, biomechanical studies, mRNA, and protein analyses and analyzed the data. RS provided the expertise on mechanical testing design and analysis, NK-A helped perform the mechanical testing. EC-K designed the experiments and performed the mRNA and protein analyses. NL performed data analysis. RDP conceived and designed the study and prepared the manuscript. All authors contributed to the article and approved the submitted version.

## Supplementary material

Supplementary Figure 1 Body weight determined at age 2, 7, 12 months in: **(A).** female *Sirt1^+/Δ^* and WT mice **(B).** male *Sirt1^+/Δ^* and WT mice. Results are Mean ± SEM analyzed by unpaired Student’s *t*-test; ***P*<0.01 versus WT mice (n=7-11 mice/group)

Supplementary Figure 2 Femoral length determined at age 2, 7, 12 months in: **(A)**. female *Sirt1^+/Δ^* and WT mice **(B).** male *Sirt1^+/Δ^* and WT mice. Results are Mean ± SEM analyzed by unpaired Student’s *t*-test; ***P*<0.01 and *****P*<0.0001 versus WT female mice.
