# Supplementary figures and images for: SIRT1 haplo-insufficiency results in reduced cortical bone thickness, increased porosity and decreased estrogen receptor alpha in bone in adult 129/Sv female mice
Source: Front Endocrinol (Lausanne). 2022 Dec 7;13:1032262. doi: 10.3389/fendo.2022.1032262 (PMC9768543; doi:10.3389/fendo.2022.1032262)

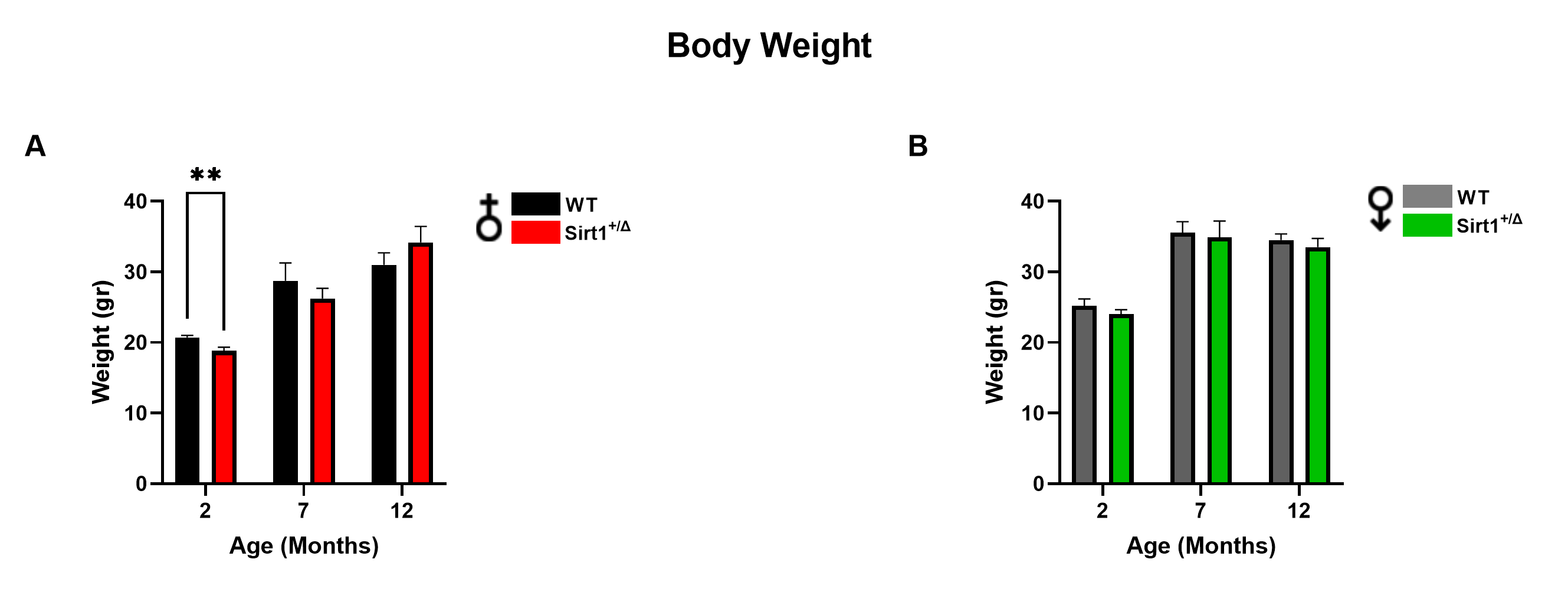

Supplement: Supplementary Figure 1 — Body weight determined at age 2, 7, 12 months in: (A). female Sirt1+/Δ and WT mice (B). male Sirt1+/Δ and WT mice. Results are Mean ± SEM analyzed by unpaired Student’s t-test; **P<0.01 versus WT mice (n=7-11 mice/group) [file Image_1.tif]

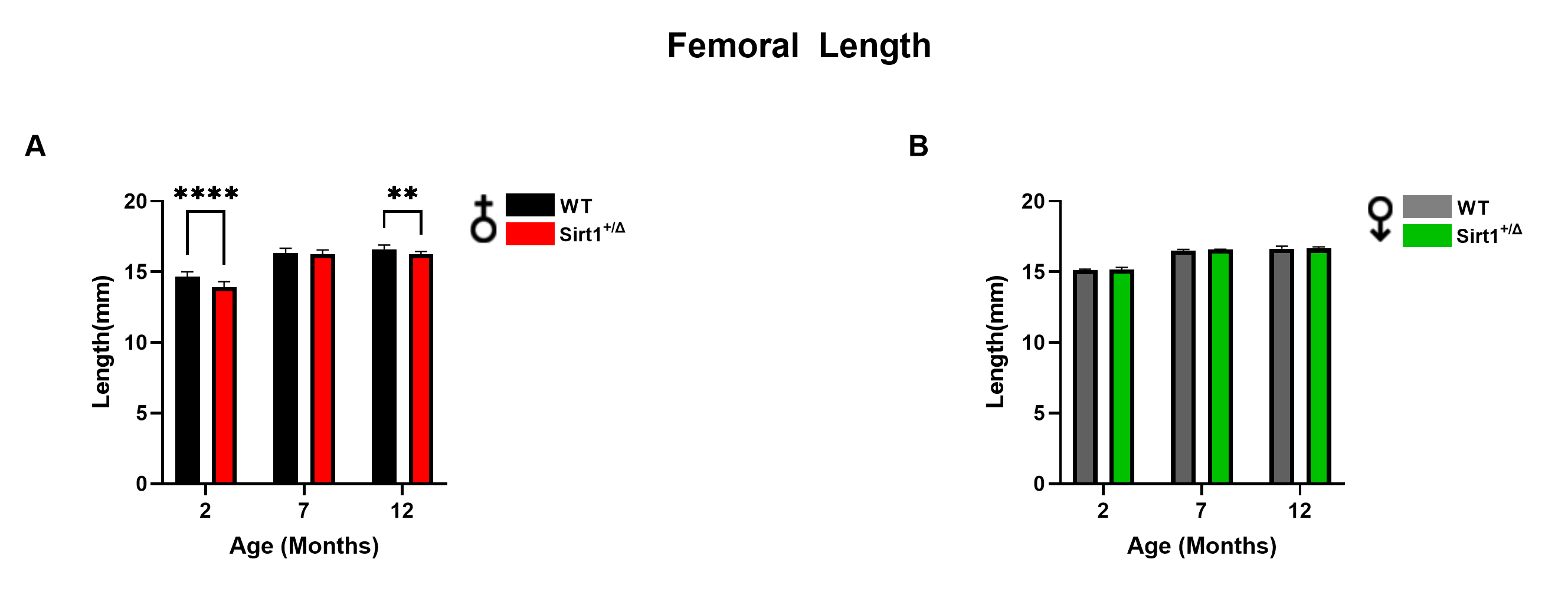

Supplement: Supplementary Figure 2 — Femoral length determined at age 2, 7, 12 months in: (A). female Sirt1+/Δ and WT mice (B). male Sirt1+/Δ and WT mice. Results are Mean ± SEM analyzed by unpaired Student’s t-test; **P<0.01 and ****P<0.0001 versus WT female mice. [file Image_2.tif]
